# EBR-5, a Novel Variant of Metallo-β-Lactamase EBR from Multidrug-Resistant Empedobacter stercoris

**DOI:** 10.1128/spectrum.00039-23

**Published:** 2023-01-31

**Authors:** Pei Li, Ting Lei, Yang Zhou, Yujie Dai, Zhishuang Yang, Hongyan Luo

**Affiliations:** a College of Veterinary Medicine, Southwest University, Beibei, Chongqing, China; b Research Center of Avian Diseases, College of Veterinary Medicine, Sichuan Agricultural University, Chengdu, Sichuan, China; Veterans Affairs Northeast Ohio Healthcare System

**Keywords:** *E. stercoris*, carbapenemase, EBR-5, novel variant

## Abstract

A novel chromosome-encoded metallo-β-lactamase (MBL) EBR variant, namely, EBR-5, was identified in a multidrug-resistant Empedobacter stercoris strain SCVM0123 that was isolated from chicken anal swab samples. EBR-5 shared 82.13% amino acid identity with the previously known EBR-1. The expression of EBR-5 in Escherichia coli reduced susceptibility to expanded-spectrum cephalosporins and carbapenems. Compared with *bla*_EBR-1_, the recombinant strain harboring *bla*_EBR-5_ exhibited higher minimum inhibitory concentrations of piperacillin, cefotaxime, and meropenem. Despite the genetic diversity, EBR-5 and EBR-1 possessed similar kinetic parameters, except for cefepime, cefotaxime, cefoxitin, cephalothin, and meropenem, which were hydrolyzed more by EBR-5. In addition to *bla*_EBR-1_, a whole-genome sequencing analysis of SCVM0123 also revealed a plasmid-mediated *bla*_RAA-1_ gene. This study underlines the importance of *E. stercoris* monitoring, as it could be a potential reservoir of these β-lactamase genes.

**IMPORTANCE** Carbapenemases are one of the greatest threats to clinical therapy, as they could confer resistance by hydrolyzing carbapenems and other β-lactam antimicrobials. In this study, we identified a novel metallo-β-lactamase EBR variant, namely, EBR-5, in Empedobacter stercoris. The biochemical properties, substrate hydrolysis abilities, and inhibition profiles of EBR-5 were reported. Through whole-genome sequencing and bioinformatic analyses, we revealed for the first time that the ESBL gene *bla*_RAA-1_ was located on a plasmid. This study extends the database of class B metallo-β-lactamases. Meanwhile, *E. stercoris* could be a major reservoir of *bla*_EBR-5_ and *bla*_RAA-1_, which have potential to spread to pathogens.

## OBSERVATION

Empedobacter stercoris (*E. stercoris*), a Gram-negative, rod-shaped bacterium that belongs to the *Weeksellaceae* (formerly classified as *Flavobacteriaceae*) family (1). It is usually recovered from fecal samples, and it has been reported as a major reservoir of the tigecycline resistance gene *tet*(X14) (2). However, few publications report its phenotype or its resistance mechanism toward β-lactams in *E. stercoris*.

Carbapenems are the latest developed β-lactams, and they possess a broad spectrum of activity, being frequently used to treat infections that are caused by multidrug-resistant pathogens (3). Resistance to carbapenems brings great threats to clinical therapy. The major resistance mechanism is carbapenemase, which could be found in the β-lactamases of classes A, B, and D. Several class B carbapenemases have been reported from bacteria of the *Weeksellaceae* family, including BlaB and GOB from Elizabethkingia meningoseptica (4, 5), IND from Chryseobacterium indologenes (6), CGB-1 from Chryseobacterium gleum (7), ORR-1 from Ornithobacterium rhinotracheale (8), CPS-1 from Chryseobacterium piscium (9), ESP-1 from Epilithonimonas tenax (9), and EBR from E. brevis or E. falsenii (10). Currently, the EBR variants have already been extended from EBR-1 to EBR-4 (http://bldb.eu/BLDB.php?prot=B1). In this study, we report a novel, chromosomally-located *bla*_EBR_ variant, namely. *bla*_EBR-5_, in a livestock-associated *E. stercoris* strain.

A carbapenem-resistant *E. stercoris* SCVM0123 was isolated from chicken anal swab samples that were obtained in Chongqing, China, in 2019. Antimicrobial susceptibility tests were performed via a modified Clinical & Laboratory Standards Institute (CLSI)-based method (11). The minimum inhibitory concentration (MIC) results showed that SCVM0123 was resistant to almost all of the tested antibiotic categories, including aminoglycosides, phenicols, fluoroquinolones, macrolides, lincosamides, tetracyclines, expanded-spectrum cephalosporins, and carbapenems (Table 1). To understand the resistance mechanism, the complete genome of SCVM0123 was sequenced using an Illumina HiSeq 4000 system and a PacBio RS II platform. Genome annotation and gene prediction were performed using the National Center for Biotechnology Information (NCBI) Prokaryotic Genome Annotation Pipeline (PGAP) (12). The sequencing results indicated that the genome of SCVM0123 consisted of a circular chromosome (3.03 Mb; guanine-cytosine [GC] content of 31.76%) and three plasmids: pLPY01 (10,226 bp; GC content of 34%), pLPY02 (15,774 bp; GC content of 30%), and pLPY03 (20,509 bp; GC content of 32%). A whole-genome sequencing (WGS) analysis based on NCBI’s AMRFinderPlus (13) revealed 15 putative resistance genes, more than those that were previously reported in *E. stercoris* strain ES183 (2) (Table S1). Furthermore, these resistance genes were correlated with the antibiotic resistance profiles of SCVM0123. Among these, the most prominent genes were those encoding β-lactamases, including the plasmid-mediated extended-spectrum β-lactamase (ESBL) *bla*_RAA-1_ and a novel, chromosomally-located, metallo-β-lactamase (MBL) *bla*_EBR_-like gene.

**TABLE 1:** MIC values of the antibiotics tested in this study

Antibiotic	MIC (mg/L) for strain		
	*E. stercoris* SCVM0123	E. coli BL21 (pET24a-*bla*_EBR-5_)^*a*^	E. coli BL21 (pET24a)^*a*^
Chloramphenicol	256	—^*b*^	—
Ciprofloxacin	128	—	—
Erythromycin	256	—	—
Florfenicol	256	—	—
Lincomycin	>512	—	—
Tetracycline	64	—	—
Tigecycline	8	—	—
Streptomycin	32	—	—
Amoxicillin	64	256	1
Amoxicillin-Clavulanic acid^*c*^	32	256	1
Ampicillin	64	256	0.5
Ampicillin-Sulbactam^*d*^	32	256	0.5
Aztreonam	256	≤0.125	≤0.125
Benzylpenicillin	64	256	4
Cefepime	16	0.5	≤0.125
Cefotaxime	128	8	≤0.063
Cefotaxime-Clavulanic acid^*c*^	4	8	≤0.031
Cefoxitin	4	2	1
Ceftazidime	64	1	≤0.125
Ceftiofur	64	4	≤0.125
Cefuroxime	128	32	0.5
Cephalothin	256	32	2
Cefradine	256	128	8
Doripenem	1	0.5	0.031
Ertapenem	2	0.5	≤0.016
Imipenem	0.25	0.5	0.063
Meropenem	4	2	0.031
Piperacillin	128	32	0.5
Piperacillin-Tazobactam^*e*^	64	32	0.5

a Kanamycin (50 mg/L) and IPTG (0.2 mM) were fixed when the MIC values were tested.

b *—*, no test.

c Clavulanic acid was fixed at a concentration of 2 mg/L.

d Sulbactam was fixed at a concentration of 4 mg/L.

e Tazobactam was fixed at a concentration of 4 mg/L.

The *bla*_RAA-1_ was recently identified in the genome of Riemerella anatipestifer RCAD0122, and it conferred high-level resistance to extended-spectrum cephalosporins (14). At present, this gene was not found in any clinical pathogens, except for RCAD0122. However, in this study, we discovered that it was located on the pLPY03 plasmid of *E. stercoris* SCVM0123. The GC content of *bla*_RAA-1_ (32%) was the same as that of pLPY03, but it was slightly different from that of the RCAD0122 genome (35.03%). An analysis of the genetic environment revealed that the SCVM0123-harbored *bla*_RAA-1_ gene was embedded in a putative composite transposon in pLPY03 that was bracketed by two copies of IS982 family insertion sequences (IS) (Fig. S1). When the whole sequence of pLPY03 was aligned to the approximately 20 kb region of RCAD0122 that harbored the *bla*_RAA-1_ gene, the results indicated that only the *bla*_RAA-1_ region that was flanked by IS had 100% identity (14). As *E. stercoris* and R. anatipestifer possessed the same transposon structure, we speculate that the putative composite transposon has contributed to the dissemination of the *bla*_RAA-1_ gene among these strains. Moreover, the *bla*_RAA-1_ genes that were found in these strains might be all from a common origin.

**FIG 1:**
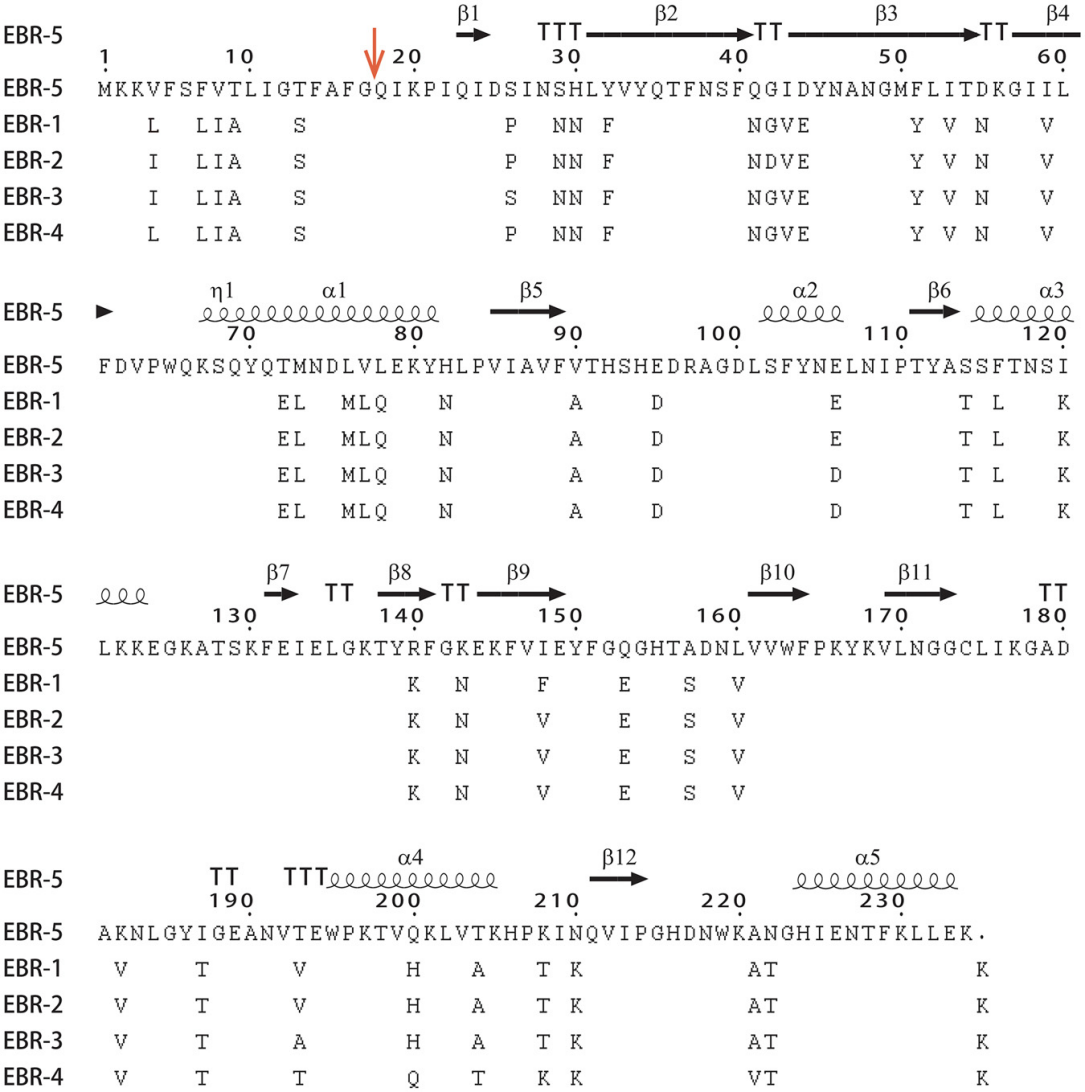
Amino acid sequence alignment of EBR-5 with other EBR alleles using Clustal Omega and ESPript 3.0. The secondary structure elements of EBR-5 are represented above the sequences. A red vertical arrow indicates the putative cleavage site of the peptide leader for EBR-5. The origin of the EBRs (GenBank accession numbers) are as follows: EBR-1 from *E. brevis* ASS-1 (AAN32638), EBR-2 from *E. falsenii* Wf 282 (ALG03771), EBR-3 from *E. falsenii* 174820 (QEJ74008), EBR-4 from *E. falsenii* Q1655 (QHT72954), and EBR-5 from *E. stercoris* SCVM0123 (UWX67704).

**FIG 2:**
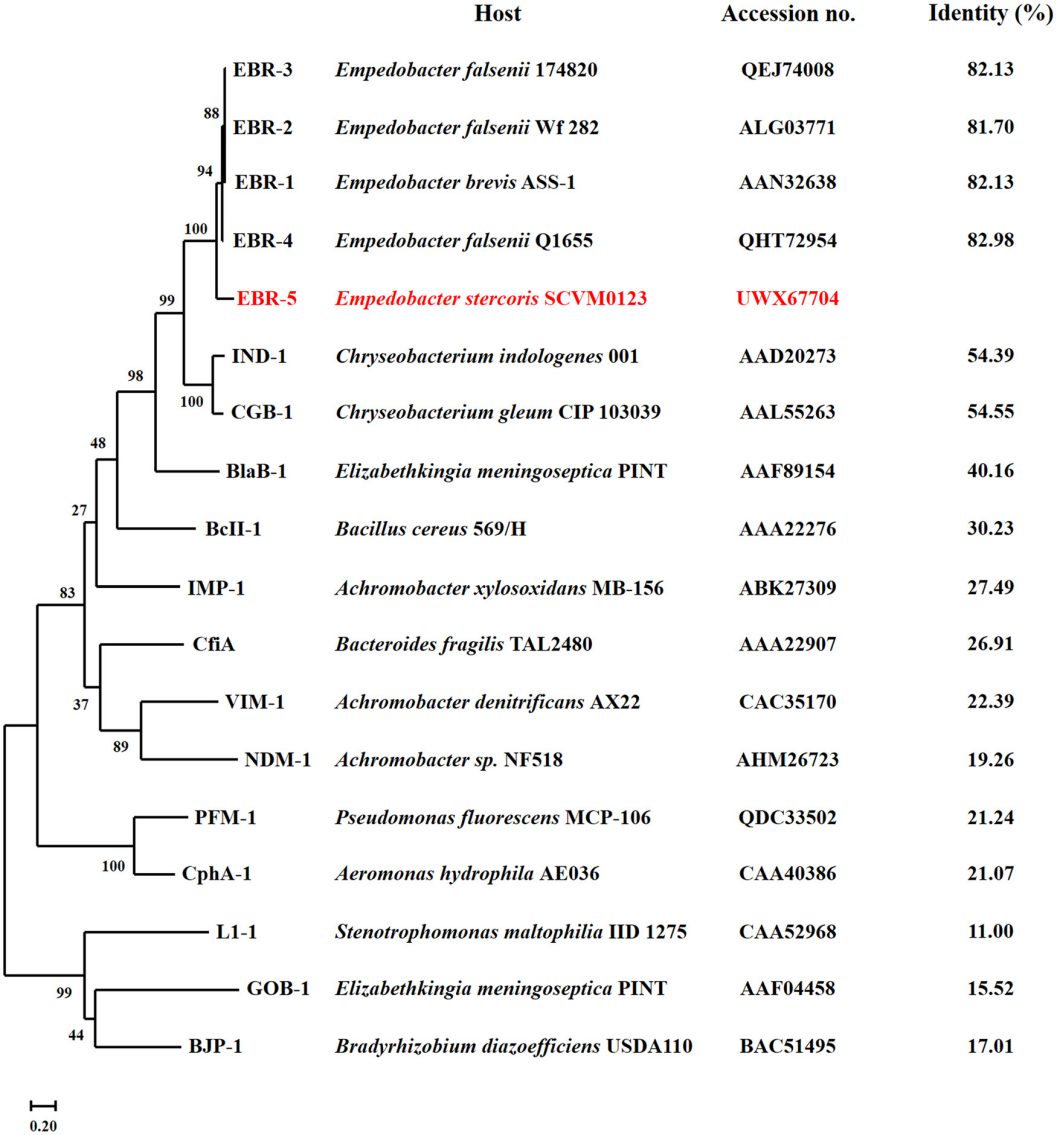
Phylogenetic analysis of the amino acid sequences of EBR-5 with those of other EBR variants and representative class B MBLs. The maximum-likelihood tree was inferred using MEGA X with 1,000 bootstrap replicates. The host strains, accession numbers, and identities of each β-lactamase, relative to EBR-5 (in red), are listed.

The WGS and polymerase chain reaction (PCR)-confirmed sequence both revealed a novel *bla*_EBR_-like gene. This gene was located at positions 786783 to 787487 on the genome of SCVM0123 (CP104209, locus_tag NZD85_03605), encoding a 234-amino-acid protein (protein_id: UWX67704). The results of an amino acid sequence alignment that was performed using the NCBI Basic Local Alignment Search Tool (BLAST) showed that 7 hits were obtained with an identity of >94% and a coverage of 100%, including 1 amino-acid sequence, 2 complete genome sequences, and 4 draft genome sequences (Table S2) (accessed 20 September 2022). The alignment results also showed that this protein barely exceeded 80% identity to the previously reported EBR alleles EBR-1 to EBR-4 (which are nearly identical to each other) (15). The apparent amino acid sequence changes indicated that this MBL enzyme was a new variant, and as such, it was designated EBR-5 (Fig. 1). Interestingly, EBR-5 was exclusively detected in *E. stercoris*. A phylogenetic analysis showed that EBR-5 belonged to Ambler subclass B1 MBL and that it was branched separately from the previously reported EBR variants (Fig. 2). The cleavage site for the leader peptide of EBR-5 was predicted to be located between positions 17 and 18 (AFG-QI) (Fig. 1).

To characterize the function of *bla*_EBR-5_, the intact gene was cloned into pET24a and transformed into E. coli BL21(DE3) (Table S3). Compared with a negative control, a 2- to 512-fold increase in the MIC was observed for the *bla*_EBR-5_ recombination clones, except for aztreonam, suggesting that *bla*_EBR-5_ was active against penicillins, cephalosporins, cephamycins, and carbapenems (Table 1). The MICs of β-lactams were not lowered by the addition of clavulanic acid, sulbactam, or tazobactam, indicating that these inhibitors could not inhibit the β-lactams’ hydrolyzing activity of EBR-5. Compared with the previously reported EBR-1 (10), EBR-5 exhibited similar susceptibility to β-lactams, with the exceptions of piperacillin, cefotaxime, and meropenem, which were 4-, 16-, and 8-fold decreased, respectively. These results indicated that EBR-5 was a carbapenemase and conferred resistance to expanded-spectrum cephalosporins and carbapenems.

For the purification of native EBR-5, the *bla*_EBR-5_ gene, without its N-terminal periplasmic signal peptide, was cloned into pMAL2 and transferred into E. coli ER2566 (insoluble expression of EBR-5 in pET24a, data not shown). The protein purification and kinetic measurements were performed as described previously (10, 16). Using a β-lactamase activity assay, EBR-5 exhibited a low-micromolar affinity for cefotaxime, cefuroxime, cephalothin, and nitrocefin (*K_m_* < 65 μM), which was similar to that of EBR-1 (10), IND-2 (17), and GOB-1 (5). The catalytic efficiency values (*k*_cat_/*K_m_*) of EBR-5 for ampicillin, benzylpenicillin, cephalothin, and nitrocefin were high (*k*_cat_/*K_m_* > 400 s^−1^ · mM^−1^). Meanwhile, EBR-5 had high *k*_cat_ values for all tested carbapenems (*k*_cat_ > 120 s^−1^) with high catalytic efficiency (*k*_cat_/*K_m_* > 100 s^−1^ · mM^−1^). In addition, the hydrolysis spectrum of EBR-5 included cefepime, cefoxitin, and ceftazidime, despite low catalytic efficiencies. Compared with EBR-1, the catalytic efficiency of EBR-5 was lower for benzylpenicillin, cefuroxime, and imipenem, but it was higher for cefepime, cefotaxime, cefoxitin, cephalothin, and meropenem (10). However, as is the case with other MBLs, EBR-5 had no detectable affinity for aztreonam (Table 2) (10, 17). Similarly, as with other zinc-dependent β-lactamases (10), the hydrolytic activity of EBR-5 was inhibited by EDTA (IC_50_, 130 μM) but not by clavulanic acid (IC_50_ > 2 mM). These kinetic parameters showed that EBR-5 was a novel variant of EBR carbapenemase.

**TABLE 2:** Kinetic parameters of EBR-5 from E. coli ER2566 compared with those of EBR-1 from *E. brevis* clinical isolate ASS-1 (10)

β-lactam	EBR-5			EBR-1		
	*K*_cat_ (s^−1^)	*K_m_* (μM)	*K*_cat_/*K_m_* (s^−1^ · mM^−1^)	*K*_cat_ (s^−1^)	*K_m_* (μM)	*K*_cat_/*K_m_* (s^−1^ · mM^−1^)
Ampicillin	118 ± 3.3^*a*^	207 ± 21	572	—^*b*^	—	—
Aztreonam	ND^*c*^	ND	ND	<0.1	ND	ND
Benzylpenicillin	86 ± 2.8	116 ± 16	745	115	47	2,500
Cefepime	0.1 ± 0.01	1,058 ± 58	0.1	<0.01	ND	ND
Cefotaxime	2.8 ± 0.1	29 ± 3.9	94	1	50	20
Cefoxitin	1.5 ± 0.04	182 ± 12	8.5	0.2	140	2
Ceftazidime	0.9 ± 0.09	567 ± 76	1.6	>2.5	>1,000	2
Cefuroxime	2.1 ± 0.07	19 ± 2.3	111	0.5	1	350
Cephalothin	27 ± 0.8	64 ± 6.4	419	6	31	200
Nitrocefin	18 ± 0.5	15 ± 1.7	1,172	—	—	—
Imipenem	124 ± 10	1,218 ± 147	102	190	782	250
Doripenem	145 ± 14	1,283 ± 161	113	—	—	—
Ertapenem	151 ± 13	1,507 ± 175	100	—	—	—
Meropenem	147 ± 8.3	626 ± 61	236	>190	>2,000	100

a ±, the errors shown are standard errors so as to fit the data to Michaelis-Menten kinetics.

b —, not available.

c ND, not detectable activity.

In summary, this study reports the discovery of a novel, chromosomally-located *bla*_EBR-5_ and a plasmid-mediated *bla*_RAA-1_ in a multidrug-resistant *E. stercoris* strain SCVM0123. Our study extends the database of class B carbapenemases and highlights the need to monitor *E. stercoris*, as it might play an essential role in the dissemination of β-lactamase genes.

### Data availability.

The complete sequences of the *E. stercoris* SCVM0123 chromosome and plasmids (pLPY01, pLPY02, and pLPY03) were deposited at GenBank under the accession numbers CP104209 to CP104212.
