# Supplementary material for: EBR-5, a Novel Variant of Metallo-β-Lactamase EBR from Multidrug-Resistant Empedobacter stercoris
Source: Microbiol Spectr. 2023 Jan 31;11(2):e00039-23. doi: 10.1128/spectrum.00039-23 (PMC10101081; doi:10.1128/spectrum.00039-23)
Supplement: Supplemental file 1 — Fig. S1. Download spectrum.00039-23-s0001.pdf, PDF file, 0.3 MB [file spectrum.00039-23-s0001.pdf]

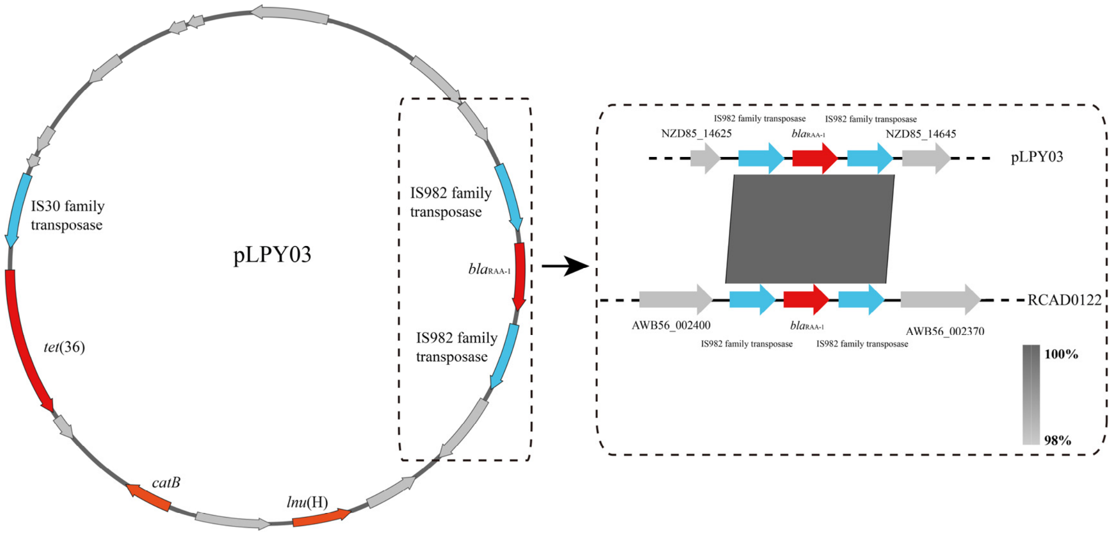

Supplementary Figure S1 Genetic environment of the *bla*RAA-1 gene in *E. stercoris* SCVM0123 and *R. anatipestifer* RCAD0122. Sequence comparison of pLPY03 plasmid and the ~20 kb region of RCAD0122 that harbored *bla*RAA-1 gene by MAUVE version 2.4.0. The arrows represent the positions and transcriptional directions of ORFs. Genes are color-coded, depending on their functional annotations: red, antimicrobial resistance gene; blue, putative transposase; light grey, other functions or hypothetical protein.
