# Supplementary material for: EBR-5, a Novel Variant of Metallo-β-Lactamase EBR from Multidrug-Resistant Empedobacter stercoris
Source: Microbiol Spectr. 2023 Jan 31;11(2):e00039-23. doi: 10.1128/spectrum.00039-23 (PMC10101081; doi:10.1128/spectrum.00039-23)
Supplement: Supplemental file 2 — Table S1. Download spectrum.00039-23-s0002.pdf, PDF file, 0.3 MB [file spectrum.00039-23-s0002.pdf]

Supplementary Table S1 Resistant genes were detected in *E. stercoris* SCVM0123 by NCBI's AMRFinderPlus.

Protein identifier	Gene symbol	Class	Location	Length	Reference protein name	Reference protein ID	% Coverage of reference sequence	% Identity to reference sequence
UWX67704	$bla_{\text{EBR-5}}$	β-Lactamase	chromosome	234	EBR-4	WP_114999921.1	99.57	83.33
UWX68213	*tet* (X2)	Tetracycline	chromosome	388	Tet (X2)	WP_008651082.1	100	100
UWX68214	*tet* (X2)	Tetracycline	chromosome	388	Tet (X2)	WP_008651082.1	100	100
UWX66004	*sul2*	Sulfonamide	chromosome	271	Sul2	WP_001043260.1	100	100
UWX66216	*tet* (X4)	Tetracycline	chromosome	383	Tet (X4)	WP_094309310.1	98.96	95.8
UWX66218	*erm* (F)	Macrolide	chromosome	266	Erm (F)	WP_002682030.1	100	99.25
	bla_{OXA}	β-Lactamase	chromosome	209	OXA-347	WP_004295324.1	74.82	99.02
UWX66222	*aadS*	Aminoglycoside	chromosome	287	AadS	WP_003013318.1	100	100
UWX66224	*tet* (X2)	Tetracycline	chromosome	388	Tet (X2)	WP_008651082.1	100	100
UWX68437	*floR*	Phenicol	Plasmid pLPY01	407	FloR	WP_000214122.1	99.01	99.75
UWX68438	*aadS*	Aminoglycoside	Plasmid pLPY01	287	AadS	WP_003013318.1	100	100
UWX68478	$bla_{\text{RAA-1}}$	β-Lactamase	Plasmid pLPY03	290	RAA-1	WP_061710074.1	100	100
UWX68463	*lnu*(H)	Lincosamide	Plasmid pLPY03	256	Lnu (H)	WP_160464899.1	100	94.53
UWX68465	*catB*	Phenicol	Plasmid pLPY03	209	CatB3	WP_032492194.1	97.62	73.17
UWX68467	*tet* (36)	Tetracycline	Plasmid pLPY03	639	Tet (36)	WP_006744283.1	99.53	92.94
