# Supplementary material for: EBR-5, a Novel Variant of Metallo-β-Lactamase EBR from Multidrug-Resistant Empedobacter stercoris
Source: Microbiol Spectr. 2023 Jan 31;11(2):e00039-23. doi: 10.1128/spectrum.00039-23 (PMC10101081; doi:10.1128/spectrum.00039-23)
Supplement: Supplemental file 3 — Table S2. Download spectrum.00039-23-s0003.pdf, PDF file, 0.2 MB [file spectrum.00039-23-s0003.pdf]

Supplementary Table S2 Strains harboring *bla*$_{EBR-5}$-like gene in GenBank.

Host	GenBank accession no.	Gene			Protein		
		Locus_tag	Coverage	Identity	Accession no.	Coverage	Identity
E. stercoris SCVM0123	CP104209	NZD85_03605	100%	100%	UWX67704	100%	100%
E. stercoris ES183	CP053698	HNV03_06740	100%	97.45%	QNT14384	100%	97.45%
E. stercoris ES202	JABFOQ010000001	HMH06_00420	100%	97.59%	NOJ74320	100%	97.87%
E. stercoris ES215	JACXZC010000005	IF125_04090	100%	97.59%	MCA4781443	100%	97.87%
E. stercoris ES180	JACXZB010000002	IF076_01415	100%	94.05%	MCA4775738	100%	94.02%
E. stercoris ES182	JACXZR010000001	IF128_00455	100%	97.45%	MCA4808242	100%	97.44%
uncultured bacterium					QQW39435	100%	98.29%
