# Supplementary material for: EBR-5, a Novel Variant of Metallo-β-Lactamase EBR from Multidrug-Resistant Empedobacter stercoris
Source: Microbiol Spectr. 2023 Jan 31;11(2):e00039-23. doi: 10.1128/spectrum.00039-23 (PMC10101081; doi:10.1128/spectrum.00039-23)
Supplement: Supplemental file 4 — Table S3. Download spectrum.00039-23-s0004.pdf, PDF file, 0.3 MB [file spectrum.00039-23-s0004.pdf]

Supplementary Table S3 Strains, plasmids and primers used in this study.

Strains or plasmid	Description	Source or reference
**Strain**		
E. stercoris SCVM0123	*E. stercoris* isolate from chicken anal swab samples obtained in 2019, conferring penicillins, cephalosporins, cefoxitin and carbapenems resistant	This study
E. coli BL21 (DE3)	F^- *ompT gal dcm lon* $hsdS_B$ ($r_B^- m_B^-$) λ (DE3 [*lacI lacUV5-T7p07 ind1 sam7 nin5*]) [$malB^+$] $_{K\text{-}12}$(λ^S)	NEB
E. coli ER2566	F^- λ^- *fhuA2* [*lon*] *ompT lacZ*::T7*.1 gal sulA11* Δ(*mcrC-mrr*)*114*::IS*10* R (*mcr-73*::miniTn*10*) (Tet^S)2 R(*zgb-210*::Tn*10*) (Tet^S) *endA1* [*dcm*]	NEB
**Plasmid**		
pET24a	pBR322, T7/*lacI*, Kan^R, IPTG inducible	Lab stored
pET24a-$bla_{\text{EBR-5}}$	$bla_{\text{EBR-5}}$ from *E. stercoris* SCVM0123 genome was cloned into pET24a, Kan^R	This study
pMAL2	Derived from pMAL-c6T, pBR322, P_{tac}/*lacI*, Cm^R, IPTG inducible,	(1)
pMAL2-$bla_{\text{EBR-5}}$	$bla_{\text{EBR-5}}$ from *E. stercoris* SCVM0123 genome was cloned into pMAL2, Cm^R	This study

**Primer**			
Primers for construction of recombinant plasmids			
pET24a-$bla_{\text{EBR-5}}$	$bla_{\text{EBR-5}}$	P1	5′ ACTTTAAGAAGGAGATATACATATGAAAAAAGTATTTTCTTTTGTCAC 3′
		P2	5′ AACTCAGCTTCCTTTCGGGCTTTGTTATTTTTCTAAAAGTTTAAACG 3′
	pET24a	P3	5′ ACAAAGCCCGAAAGGAAGC 3′
		P4	5′ CATATGTATATCTCCTTCTTAAAGTTAAAC 3′
pMAL2- $bla_{\text{RAA-1}}$	$bla_{\text{EBR-5}}$	P5	5′ ATTGACCAACAAGGACCATAGATTATGCAAATAAAACCGATTCAAATAG 3′
		P6	5′ TCGACGATATCGCGGCCGCCCATCAGTTATTTTTCTAAAAGTTTAAACG 3′
	pMAL2	P7	5′ TAACTGATGGGCGGCCGCGATATCG 3′
		P8	5′ CATAATCTATGGTCCTTGTTGGTCAATTG 3′

References

1. Luo H, Zhu D, Li M, Tang Y, Zhang W, Wang H, Li P. 2022. RAA Enzyme Is a New Family of Class A Extended-Spectrum beta-Lactamase from *Riemerella anatipestifer* Strain RCAD0122. Antimicrob Agents Chemother 66 (3): e0175721. http://doi.10.1128/AAC.01757-21.
